# Efficacy of arch contouring foot orthoses for midfoot osteoarthritis: Protocol for a randomised controlled trial

**DOI:** 10.1002/jfa2.70000

**Published:** 2024-09-02

**Authors:** Polly Q. X. Lim, Hylton B. Menz, Karl B. Landorf, Michelle R. Kaminski, Andrew K. Buldt, Merridy J. Lithgow, Kade L. Paterson, Jill Halstead, Shannon E. Munteanu

**Affiliations:** ^1^ Discipline of Podiatry School of Allied Health, Human Services and Sport La Trobe University Melbourne Victoria Australia; ^2^ Department of Podiatry Monash Health Melbourne Victoria Australia; ^3^ School of Primary and Allied Health Care Monash University Melbourne Victoria Australia; ^4^ Department of Physiotherapy Centre for Health, Exercise and Sports Medicine School of Health Sciences The University of Melbourne Melbourne Victoria Australia; ^5^ University of Leeds NIHR Leeds Biomedical Research Centre, and Leeds Community Healthcare NHS Trust Leeds UK

**Keywords:** clinical trial protocol, foot, foot orthoses, midfoot, orthotic devices, osteoarthritis, randomised controlled trial

## Abstract

**Introduction:**

Midfoot osteoarthritis (OA) is a painful and disabling condition. Arch contouring foot orthoses have been recommended for midfoot OA, yet there is no high‐quality evidence from randomised controlled trials to support their use. This clinical trial aims to evaluate the efficacy of arch contouring foot orthoses for midfoot OA.

**Methods:**

This will be a parallel‐group randomised controlled superiority trial. One‐hundred and forty community‐dwelling people with painful midfoot OA will be randomised to receive either arch contouring foot orthoses or flat sham inserts. Outcome measures will be obtained at baseline, 4, 8 and 12 weeks; the primary endpoint for assessing efficacy being 12 weeks. The primary outcome measure will be average midfoot pain whilst walking over the last 7 days on an 11‐point numerical rating scale. Secondary outcome measures include function (walking/standing subscale of the Manchester–Oxford Foot Questionnaire), participants' perception of overall treatment effect (self‐reported global rating of change on a 15‐point Likert scale), physical activity (Incidental and Planned Exercise Questionnaire), general health‐related quality of life (Short Form‐12 Version® 2.0), use of co‐interventions and adverse events.

**Discussion:**

This trial will evaluate the efficacy of arch contouring foot orthoses for relieving pain and improving function, physical activity and health‐related quality of life in people with midfoot OA. The findings will provide high‐quality evidence as to whether arch contouring foot orthoses are efficacious and will help to inform clinical guidelines about the use of foot orthoses for midfoot OA.

**Trial registration:**

Australian and New Zealand Clinical Trial Registry (ACTRN12623000953639).

## 1 INTRODUCTION

Midfoot osteoarthritis (OA) is a common and painful condition that affects 1 in 8 adults over the age of 50 [1]. The midfoot is made up of several articulations including the tarsometatarsal joints (first, second and third cuneo‐metatarsal joints [CMJs] and fourth and fifth cuboid‐metatarsal joints), the navicular‐first, ‐second and ‐third cuneiform joints (N1CJ, N2CJ and N3CJ), the talonavicular joint (TNJ) and the calcaneocuboid joint [2, 3, 4, 5, 6]. Although midfoot OA is often described as dorsal midfoot pain aggravated by weight‐bearing activities [7], there is no universally agreed definition or diagnostic criteria for this condition [4, 8, 9]. Midfoot OA typically affects more than one joint, and three phenotypes have been reported based on the pattern of joint involvement observed in radiographs: the medial midfoot (TNJ, N1CJ or first CMJ), the central midfoot (second CMJ) or both the medial and central midfoot joints [4]. Midfoot OA is the most disabling type of foot OA [3] and significantly impacts both physical and mental health‐related quality of life [10, 11]. People with midfoot OA experience physical impairments, including reduced foot and leg muscle strength [12], and have limitations in activities, such as walking and climbing stairs [13, 14], with over 80% reporting the condition to be disabling [14]. In addition, they report increased levels of anxiety and depression [13, 14]. The aetiology of midfoot OA is multi‐factorial and not completely understood. However, several factors are associated with the condition including older age, female sex, manual occupations, obesity, pain in other weight‐bearing joints, previous foot and ankle injury, and having other non‐musculoskeletal comorbidities [14].

Altered foot and lower limb biomechanics are also associated with midfoot OA [4, 13, 15, 16, 17]. A systematic review found that people with midfoot OA have a flatter foot posture, longer central metatarsals, greater first ray mobility, reduced range of motion in the subtalar and first metatarsophalangeal joints as well as increased peak plantar pressures, pressure time integrals and contact times in the heel and midfoot while walking [15]. These variations are hypothesised to create increased mechanical stress within the midfoot, which may contribute to the development of midfoot OA. These findings are supported by investigations using magnetic resonance imaging (MRI), which have identified patterns of bone marrow lesions, an indicator of bone stress, in the midfoot region of people with symptomatic midfoot OA [18]. Furthermore, biomechanical studies have shown an association between increased medial plantar loading of the foot with pain and disability in people with midfoot OA [13, 16].

Despite the burden of foot OA, there are no clinical guidelines for the management of midfoot OA [10, 19]. Several interventions have been recommended for the initial management of midfoot OA, including arch contouring foot orthoses [20, 21], but there is uncertainty regarding their clinical effectiveness [22]. One biomechanical study reported that arch contouring foot orthoses decreased hindfoot and forefoot maximum force and peak pressure, and increased maximum force and contact area under the midfoot [23]. These effects have been theorised to be beneficial by reducing bending moments across the midfoot joints, reducing medial longitudinal arch deformation and midfoot joint compression [16, 20, 23]. Despite this mechanistic reasoning, only two studies [20, 21] have evaluated the efficacy of arch contouring foot orthoses for midfoot OA. In a case series of 57 participants [21], the use of custom‐made arch contouring foot orthoses (made of dual‐density ethylene vinyl acetate) was associated with improved pain and function for up to 6 months. More recently, a feasibility randomised controlled trial [20] of 37 participants demonstrated improvements in foot pain and foot‐related disability with the use of prefabricated arch contouring foot orthoses (VectOrthotic® devices made of a three‐quarter composite polypropylene with a 4 mm compressed closed cell polyethylene top cover) when compared to flat inserts (4 mm compressed closed cell polyethylene) at 12 weeks. Although these findings are promising, there remains uncertainty regarding the effectiveness of arch contouring foot orthoses for midfoot OA as these studies either lacked a control group [21] or were insufficiently powered to statistically detect clinically important findings [20].

Accordingly, high‐quality randomised controlled trials that are adequately powered to detect clinically important findings are now needed. Therefore, the aim of this trial is to evaluate the efficacy of foot orthoses for midfoot OA.

## 2 MATERIALS AND METHODS

### 2.1 Study design

The *F*oot *O*rthoses for *M*idfoot *O*A (FOMO) trial will be a parallel‐group, participant‐ and assessor‐blinded, randomised controlled superiority trial over 12 weeks. The study has been registered with the Australian and New Zealand Clinical Trials Registry (ACTRN12623000953639). This study protocol has been reported using the Standard Protocol Items: Recommendations for Interventional Trials (SPIRIT) guidelines [24], and the associated checklist from the 2022 extension of the SPIRIT 2013 statement [25] is included as Additional file 1.

### 2.2 Ethical approval

Ethical approval has been obtained from the La Trobe University Human Research Ethics Committee (reference number: HEC23237). All participants will provide informed consent prior to being recruited into the trial.

### 2.3 Participants

One hundred and forty participants will be recruited. To be eligible for inclusion, participants must: (i) be aged ≥18 years, (ii) have pain in the midfoot joints (located in at least one area in a predetermined dorsal midfoot region [26]) for at least 3 months that is aggravated by weight‐bearing activities, (iii) have pain in the midfoot when walking during the last 7 days that is rated an average of at least 3 out of 10 in severity on an 11‐point numerical rating scale (NRS) (where 0 represents ‘no pain’ and 10 represents the ‘worst pain imaginable’), (iv) have radiographic evidence of midfoot OA (defined in this trial as a minimum score of 1 for either observations of joint space narrowing or osteophytes in at least one of the midfoot joints in either radiographic view) assessed using the La Trobe University Atlas of Foot OA [27], (v) be able to walk household distances (more than 50 m) without the aid of a walker, crutches or cane, (vi) be willing to regularly wear shoes that will accommodate shoe inserts, and (vii) be willing to attempt to not use co‐interventions (including pain relieving oral medications) during the study period.

Participants will not be eligible for inclusion if they: (i) have had previous ipsilateral foot surgery or are planning surgery in the next 12 weeks, (ii) have OA secondary to other issues such as major trauma, (iii) have the presence of other foot or ankle condition(s) or have pain in another location that is greater than the pain in the midfoot, (iv) have any musculoskeletal or systemic medical conditions (e.g. suspected inflammatory joint disease or neurological conditions) that could make them unsuitable for inclusion or confound pain and functional assessments of the midfoot, (v) are unable to speak and read English, (vi) have had treatment that includes the use of any foot orthoses (including shock absorption shoe inserts), ankle braces and/or, received intra‐articular injections at the midfoot in the previous 12 weeks, and (vii) have dementia or any cognitive impairment.

### 2.4 Recruitment

Participants will be recruited using the following methods: (i) postal invitation using a database of patients from the La Trobe University Health Sciences Clinic, (ii) social media advertising, (iii) posters placed in the local community, (iii) mail‐out and/or online advertisements to healthcare practitioners in the local area, and (iv) provision of information flyers at orthopaedic triage clinics in hospitals around Melbourne, Australia.

### 2.5 Baseline assessments

Participant characteristics (such as age, gender, sex, country of birth, ethnicity, education, major medical conditions, medications and foot pain duration) will be obtained upon enrolment via an online questionnaire designed in a secure web platform (REDCap™, Research Electronic Data Capture, Vanderbilt University, USA). The following anthropometric measurements and clinical assessments will be conducted in person: (i) height and weight will be measured using a digital scale and body mass index will be calculated as weight (kg)/height (m^2^), (ii) foot pain and location will be determined by clinical assessments, such as palpation and passive range of motion testing [28], and self‐reported using a standardised foot manikin diagram (^©^The University of Manchester 2000. All rights reserved) [26], (iii) static foot posture will be assessed using the Foot Posture Index [29], (iv) foot and ankle range of motion will be measured based on previously published, reliable protocols [30, 31], (v) foot and ankle muscle strength will be measured with a CITEC hand‐held dynamometer (CIT Technics, The Netherlands) using a previously documented, reliable protocol [32], and (vi) footwear will be assessed using selected items from the Footwear Assessment Tool [33].

### 2.6 Radiographic assessment

Participants will be required to undergo radiographic imaging (weight‐bearing radiographs in dorso‐plantar, lateral and oblique projections) of their symptomatic foot (or most symptomatic foot). The severity of radiographic midfoot OA will be evaluated using the La Trobe University Atlas of Foot OA [27]. The radiographs will be assessed by experienced investigators (HBM and SEM) with agreement by consensus. Radiographic evidence of midfoot OA will be defined as having a score of at least 1 for either the presence of joint space narrowing or osteophytes in any of these midfoot joints (first and second CMJ, N1CJ, or the TNJ) from any of the radiographic projections.

### 2.7 Randomisation

Participants will be randomised to either the arch contouring foot orthoses or the sham inserts in a 1:1 ratio with random permuted block sizes of 4 and 6 using an online randomisation service (www.sealedenvelope.com). An independent person who is not involved in the study design, recruitment or data analysis will create and upload the allocation sequence to REDCap™. The randomisation feature in REDCap™ facilitates allocation concealment by having different user rights. To prevent subversion of the allocation sequence, none of the assessors involved in recruitment and data collection will have user rights to access the uploaded allocation sequence. All baseline assessments will be completed prior to group allocation.

### 2.8 Blinding

Participants will be blinded to group allocation by limited disclosure. Participants will be advised that the clinical trial has been designed to compare one of two types of shoe inserts for midfoot OA, but they will not be informed about the specific characteristics of the interventions. Due to the nature of the intervention (i.e. foot orthoses), research staff administering them cannot be blinded. The interventions will be referred to as ‘shoe inserts’ throughout the study and both groups will receive the same clinical guideline‐based information and support at the start of the trial. Primary and secondary outcomes are participant‐reported, thus this trial will also be considered assessor‐blinded (as participants are blinded). Trial personnel entering participant‐reported data and the biostatistician performing the statistical analyses for the trial will be blinded.

### 2.9 Study procedure

**FIGURE 1:**
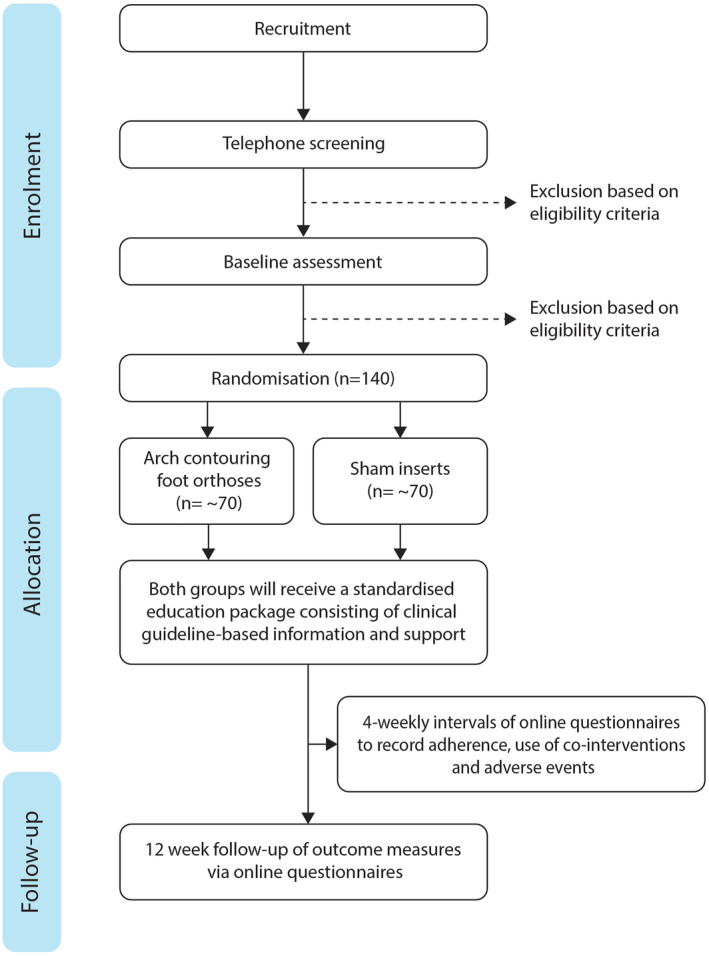
Participant flow through the trial.

Participant flow through the trial is outlined in Figure 1. All face‐to‐face assessments will be performed in the Foot and Ankle Laboratory at La Trobe University and/or the La Trobe University Health Sciences Clinic in Melbourne, Victoria, Australia. Online questionnaires via REDCap™ will be sent every 4 weeks, with the final follow‐up questionnaire at 12 weeks. Table 1 shows the schedule of enrolment, interventions that will be used and the outcome measures for this trial according to the SPIRIT recommendations [24, 25].

**TABLE 1:** SPIRIT diagram of enrolment, interventions and assessment for the FOMO trial.

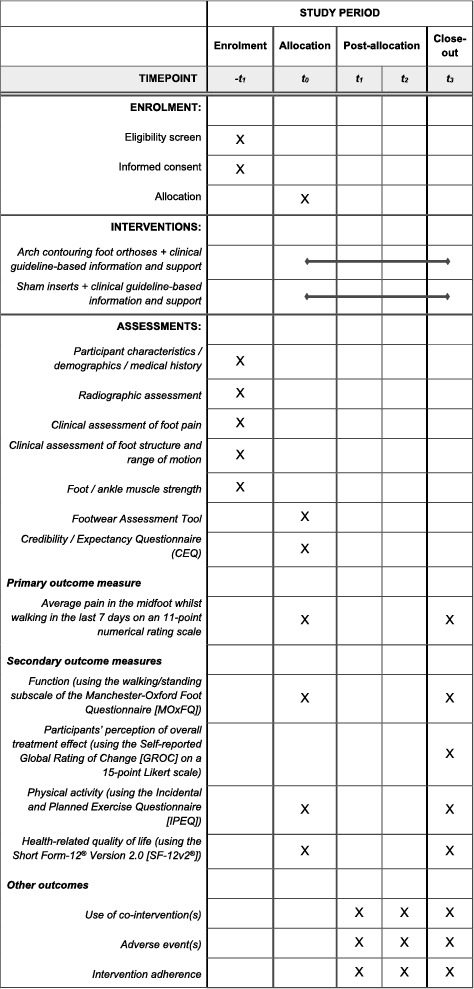

Abbreviations: FOMO, *F*oot *O*rthoses for *M*idfoot *O*steoarthritis; SPIRIT, Standard Protocol Items: Recommendations for Interventional Trials; −*t*_1_, enrolment (clinic); *t*_0_, allocation (clinic); *t*_1_, week 4 (online); *t*_2_, 8 week (online); *t*_3_, week 12 (online or in clinic).

### 2.10 Interventions

#### 2.10.1 Intervention group

**FIGURE 2:**
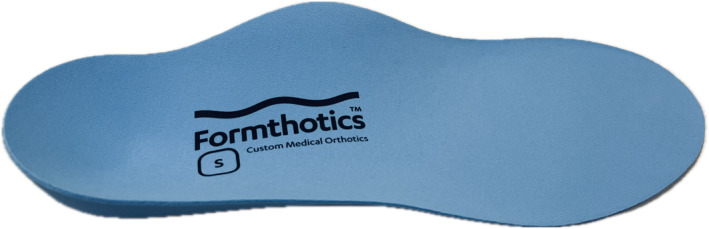
Intervention.

Participants in the intervention group will be provided with two pairs of full‐length prefabricated arch contouring foot orthoses (Formthotics™; 140 kg/m^3^ single‐density closed‐cell polyurethane foam, Foot Science International, New Zealand) (Figure 2). The foot orthoses will be fitted into the shoes that they wear most regularly by a registered podiatrist and will be modified with the addition of a 4° medium density varus wedge if the foot posture index is >7 [29, 34, 35]. As recommended by the manufacturer, the orthoses will then be heated and placed in the participant's footwear. The participant will be required to stand in their footwear with the heated foot orthoses to facilitate custom moulding to the plantar surface of the foot and medial longitudinal arch. This is the recommended standard clinical care using this intervention. The decision to use this intervention is based on our recent survey on the current practices of Australian podiatrists for the management of midfoot OA (manuscript in preparation).

#### 2.10.2 Control group

**FIGURE 3:**
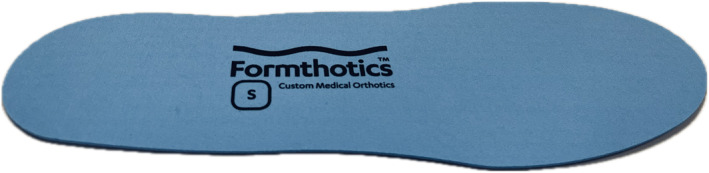
Control.

Participants in the control group will be provided with two pairs of full‐length sham inserts made from 3 mm prefabricated flat inserts (140 kg/m^3^ single‐density, closed‐cell polyurethane foam, Foot Science International, New Zealand). The sham inserts will be the same colour and have the same branding as the foot orthoses used in the intervention group but without the heel and arch support (Figure 3). This type of device is considered a credible ‘sham’, based on previous randomised trials [35, 36] that found no difference in participants' perceptions of the *credibility* of flat sham inserts and arch contouring foot orthoses, using the Credibility/Expectancy Questionnaire (CEQ) [35] and the Treatment Credibility Scale [36]. The flat sham inserts will be fitted into the shoes they wear most regularly by a registered podiatrist.

The provider of the foot orthoses and sham inserts (Foot Science International, New Zealand) will have no input into the design or implementation of the trial. They will not participate in data analysis, interpretation of the data generated from the trial, manuscript preparation or dissemination of the trial findings.

#### 2.10.3 Clinical guideline‐based information and support

Participants in both groups will receive a standardised education package derived from clinical guidelines for management of OA [37, 38] and our recent survey on the current practices of Australian podiatrists for the management of midfoot OA (manuscript in preparation). Education will include guidance regarding midfoot OA (i.e. information on epidemiology, location of affected joints and associated factors), OA flares, advice relating to exercise such as activity modification, weight loss, the use of topical non‐steroidal anti‐inflammatory drugs (NSAIDs) and footwear.

Participants in both groups will be advised to use their allocated shoe inserts in their footwear for as much time as they can during the day for the 12 weeks of the trial. Insole wear will be monitored by recording the average days per week, plus the average number of hours per day the participant has worn the arch contouring foot orthoses or sham inserts, assessed via online questionnaires at each 4‐weekly timepoint. Participants will be requested to attempt to not use co‐interventions (including pain relieving oral medications) during the study, but if they do, they are required to document the use of co‐interventions in the 4‐weekly online questionnaires.

### 2.11 Treatment credibility/expectation

Treatment credibility (participants' beliefs about the logic underpinning the intervention) and treatment expectancy (participants' perceptions of how much they may benefit) will be measured using the CEQ [39]. The CEQ will be administered after allocation. The CEQ consists of six items; three are related to credibility and three are related to expectancy. For each item, participants will be asked to rate the credibility of the intervention and their expectations on a 9‐point Likert scale. High scores on the scale indicate that the participant considers the intervention to be credible and expects it to be effective.

### 2.12 Outcome measures

The primary outcome will be measured prior to allocation at baseline and at 12 weeks (the primary endpoint). All secondary outcomes unless stated otherwise will be measured prior to allocation at baseline and at 4, 8 and 12 weeks. Participants will complete online questionnaires at 4 week intervals, and they will be encouraged to contact the researchers at any time during the trial if needed. The outcome measures and associated timepoints are shown in Table 1.

### 2.13 Primary outcome

#### 2.13.1 Pain

The primary outcome is average pain in the midfoot whilst walking in the last 7 days scored using an 11‐point NRS with terminal descriptors of 0 (‘no pain’) and 10 (‘worst pain imaginable’). This outcome measure has been used as a primary outcome measure in a feasibility trial that investigated arch contouring foot orthoses for midfoot OA [35] and has been recommended for clinical trials in OA [40]. Although there are no studies that have assessed the responsiveness of the NRS specifically in midfoot OA, the NRS has been shown to be more responsive, easier to administer and preferred by adults compared to other pain scales [41]. In people with chronic musculoskeletal pain, the minimal clinically important difference of the NRS has been reported to be between 1 and 2 points [42, 43].

### 2.14 Secondary outcomes

#### 2.14.1 Function

Function will be assessed using the walking/standing subscale of the Manchester–Oxford Foot Questionnaire (MOxFQ) [44]. The MOxFQ has been shown to be valid (content and construct), have high retest reliability (intra‐class correlation coefficient 0.96) [45] and have a high degree of internal consistency (Cronbach *α* 0.92) for the walking/standing subscale [46].

#### 2.14.2 Participants' perception of overall treatment effect

Participants' perception of overall treatment effect will be measured using the self‐reported global rating of change (GROC) scale [47] at 12 weeks. Participants will be asked: ‘With respect to your midfoot osteoarthritis, how would you describe yourself now compared to the start of the study?’, with the response measured using a 15‐point Likert scale (ranging from ‘a very great deal worse’ to ‘a very great deal better’). This outcome will then be dichotomised into the categories of ‘effective’ (‘a very great deal better’, ‘a great deal better’, ‘a good deal better’ and ‘moderately better’) and ‘ineffective’ (‘somewhat better’, ‘a little better’, ‘about the same, hardly any better at all’, ‘no change’, ‘about the same, hardly any worse at all’, ‘a little worse’, ‘somewhat worse’, ‘moderately worse’, ‘a good deal worse’, ‘a great deal worse’ and ‘a very great deal worse’) [48].

#### 2.14.3 Physical activity

Levels of physical activity will be measured using the Incidental and Planned Exercise Questionnaire (IPEQ) [49]. The IPEQ is a valid and reliable 12‐item questionnaire assessing low, basic and high‐intensity physical activities with a week recall [49].

#### 2.14.4 Health‐related quality of life

Health‐related quality of life will be measured using the Short Form‐12® Version 2.0 (SF‐12v2®) [50], a 12‐item questionnaire that is a reliable and valid measure of physical and mental health [50].

### 2.15 Use of co‐interventions

Participants will be asked to document the use of any co‐interventions at 4, 8 and 12 weeks via online questionnaires. They will be asked if they have received or used any new treatment to relieve the pain that they are experiencing in the midfoot. This includes, but is not limited to, the use of any taping or bracing, visits to healthcare providers, use of any creams/rubs or use of any medication such as anti‐inflammatory drugs. It also includes the use of topical NSAIDs and footwear changes recommended as part of the clinical guideline‐based information and support. Participants will be asked to document the type of treatment, the date(s) they received or used the treatment and if relevant, the dosage and frequency of the treatment used. Participants will also be asked if they have changed the footwear they normally wear. To minimise participant burden, the use of co‐interventions will be documented on the day with recall over the previous 4 weeks rather than daily entry surveys [51, 52, 53].

### 2.16 Evaluation of adherence

Adherence will be measured at 4, 8 and 12 weeks via online questionnaires. For both groups, participants will provide information regarding the average number of hours per day and number of days they have worn the shoe inserts (arch contouring foot orthoses or sham inserts) and their shoes in the last 4 weeks. The average time spent wearing shoe inserts when in their shoes will then be calculated [35]. Participants will be classified as adherent if they wear their shoe inserts for an average of at least 35 h per week. To minimise participant burden, adherence will be documented on the day with recall over the previous 4 weeks rather than daily entry surveys [51, 52, 53].

### 2.17 Adverse events

Adverse events relating to the use of interventions in both groups will be documented as the number of participants experiencing any adverse event (serious events and non‐serious events) and the number of participant withdrawals due to adverse events. Adverse events will be assessed at 4 week intervals via online questionnaires. Participants will be asked to document the type of adverse event, the body location, the frequency and/or the severity of the effect [48]. All reported adverse events will be reviewed by an independent assessor at the end of the trial and categorised as events that are *unrelated, probably related* or *definitely related*. Those considered to be *probably related* or *definitely related* will be considered an adverse event [54]. Serious adverse events will be defined as events that are life‐threatening, require hospitalisation or result in persistent or significant disability or incapacity [55]. If participants experience a severe adverse event (e.g. severe pain), they will be asked to contact one of the investigators.

### 2.18 Data management

Enrolment, baseline and follow‐up questionnaire data will be stored in REDCap™ and subsequently stored as electronic files on the La Trobe University secure server. Hardcopy versions of pain diagrams will be stored in a locked filing cabinet at La Trobe University and then shredded after being transferred into electronic formats for analysis (Microsoft Excel; Microsoft Corporation, USA and IBM SPSS Statistics; IBM Corporation, USA).

### 2.19 Sample size

An a priori sample size calculation was conducted using G*Power version 3.1.9.7 [56, 57]. Using an allocation ratio of 1:1, power of 80%, assuming a minimal clinically important difference of 1.1 points [20] (representing a medium effect size [Cohen's *d*] of 0.5), standard deviation of 2.2 points [20], alpha set at 0.05 and allowing a 10% participant loss to follow‐up [20], a sample size of at least 140 participants (i.e. approximately 70 per group) will be required.

### 2.20 Statistical analysis

Statistical analysis will be performed using the most recent version of SPSS available to the researchers using the intention‐to‐treat principle for all randomised participants. In participants with bilateral symptoms, the more painful side will be analysed (or the right lower limb if they cannot decide which is the more painful side) to maintain independence of data [58]. Standard tests to assess continuous data for normal distribution will be used. If continuous data is not normally distributed, transformation will be carried out if appropriate. Multiple imputation will be used to replace any missing data using five iterations, with sex, age, baseline scores and group allocation as predictors [59]. The exception will be for the GROC scale, co‐interventions, adherence and adverse events where no data substitution will be applied.

The primary outcome measure will be NRS pain measured at 12 weeks. To avoid over‐testing and to minimise the risk of type I error associated with serial measurements, statistical analysis of the efficacy of the interventions will specifically focus on the between group difference at 12 weeks [60, 61]. Differences in the primary and secondary outcome measures between the two groups will be compared. Continuously scaled outcome measures will be analysed using the analysis of covariance (ANCOVA) with baseline scores and intervention group entered as independent variables [62, 63]. Dichotomous outcome measures (e.g. frequency of adverse events, frequency of co‐interventions and dichotomised GROC) will be compared using relative risk, risk difference and number needed to treat. Intervention adherence will be compared using independent group *t*‐tests. For all analyses, both 95% confidence intervals and *p*‐values will be generated where appropriate.

A sensitivity analysis will be conducted to estimate treatment effects on the primary outcome. We will assess the robustness of the results from our analyses using the following conditions: (i) complete case analysis—only analyse participants who provide outcome data and (ii) last observation carried forward. A sensitivity analysis will also be undertaken to estimate treatment effects on the primary outcome assuming full adherence (per‐protocol analysis) [64]. Full adherence will be defined as participants reporting wearing the shoe inserts for an average of at least 35 h per week.

## 3 DISCUSSION

The aim of this randomised controlled trial is to evaluate the efficacy of arch contouring foot orthoses for midfoot OA. Our recent systematic review [22] identified no fully‐powered randomised controlled trials that had evaluated interventions for midfoot OA, so our parallel‐group superiority trial will be the first adequately powered randomised controlled trial for this condition.

Our randomised controlled trial has several strengths. Firstly, to minimise the risk of bias, we will use an external randomisation service for sequence generation and allocation concealment, and the trial methodology includes participant blinding and blinded data analysis. Secondly, we will reduce attrition by minimising the burden on participants through online data collection during follow‐up. Additionally, participants can contact the study investigators if they experience any adverse events. Thirdly, the study findings are likely to be generalisable, since the trial methods, including choice of intervention and timing of outcomes, have been informed by the findings of our recent national survey of Australian podiatrists regarding their assessment and management approaches for midfoot OA (manuscript in preparation). Further, the specific type of arch contouring foot orthoses we are using are relatively low cost and widely used in Australia (as well as other countries worldwide). Finally, the choice of outcome measures of pain, function, physical activity, health‐related quality of life and adverse events is based on expert (OMERACT: Outcome Measures in Rheumatology Initiative) guidelines [65], further enhancing the external validity of the trial findings. Therefore, outcomes from our trial will provide much‐needed generalisable evidence regarding the efficacy of a common intervention, arch contouring foot orthoses, for radiographically confirmed midfoot OA.

An important consideration of our randomised controlled trial is the diagnostic criteria that we have used for midfoot OA. There is currently no agreed‐upon diagnostic criteria for midfoot OA. Traditionally, a radiographic score of two (out of a maximum of three for osteophytes or joint space narrowing) has been considered as ‘definite OA’ [27]. However, recent guidelines from NICE suggest diagnosing OA clinically without imaging in individuals aged 45 or older who experience activity‐related joint pain [37]. Given the complexity of the midfoot, we have included both activity‐related joint pain and radiographic evidence of OA in our inclusion criteria to enhance the specificity of our diagnosis. Further, we have set the radiographic OA severity score at one, instead of two, for osteophytes or joint space narrowing to increase the sensitivity of our diagnostic criteria and to improve the generalisability of our trial findings. While we acknowledge that other sophisticated imaging techniques, such as MRI, may be more sensitive for diagnosing early stages of OA [18], they were not considered feasible in our current trial due to cost and participant burden.

One limitation of the trial is the inability to blind the research staff administering the interventions. This is recognised as a common limitation in trials using non‐pharmacologic interventions [66]. However, as the outcomes are participant‐reported and participants are blinded by limited disclosure, the study can be considered assessor‐blinded. Blinding is important to avoid overestimating effects of the intervention [67]. In addition, the most likely barrier for adherence to the intervention is the requirement of participants to wear their usual footwear. We plan to overcome this by limiting enrolment during the primary holiday period in Australia (end of December to January), where it is the height of summer.

### 3.1 Trial status

This study commenced recruitment in September 2023 and the first participant completed baseline testing on 14 September 2023. We envisage that the 12‐week follow ups of all participants will be completed by December 2024. The findings will be disseminated via peer‐reviewed journal articles and presented at national and international conferences from 2025. Further, the findings will be presented in a PhD thesis by PQXL.

## AUTHOR CONTRIBUTIONS

**Polly Q. X. Lim**: Conceptualisation; methodology; writing—original draft; writing—review & editing. **Hylton B. Menz**: Conceptualisation; methodology; supervision; writing—review & editing. **Karl B. Landorf**: Conceptualisation; methodology; supervision; writing—review & editing. **Michelle R. Kaminski**: Conceptualisation; methodology; supervision; writing—review & editing. **Andrew K. Buldt**: Methodology; writing—review & editing. **Merridy J. Lithgow**: Methodology; writing—review & editing. **Kade L. Paterson**: Methodology; writing—review & editing. **Jill Halstead**: Methodology; writing—review & editing. **Shannon E. Munteanu**: Conceptualisation; methodology; supervision; writing—review & editing.

## CONFLICT OF INTEREST STATEMENT

The authors declare that there are no known conflicts of interest related to this project that could have influenced this manuscript. AKB is a Deputy Editor of the Journal of Foot and Ankle Research (JFAR). HBM is a past Editor‐in‐Chief of JFAR. KBL and SEM are past Deputy Editors of JFAR. HBM and KBL are current Editorial Board members of JFAR as Emeritus Editors. It is a journal policy that current editors are removed from the peer review and editorial decision‐making processes for papers they have co‐authored.

## ETHICS STATEMENT

This study has obtained approval from the La Trobe University Human Research Ethics Committee (reference number: HEC23237) and all participants will provide written informed consent.

## CONSENT FOR PUBLICATION

Not applicable.

## Supporting information

Supporting Information S1

## Data Availability

On completion and publication of the study, de‐identified data may be accessed from the corresponding author upon reasonable request.
